# Daily physical activity and trajectories of care service use among older adults: the HUNT4 Trondheim 70+ study

**DOI:** 10.3389/fpubh.2025.1539179

**Published:** 2025-02-18

**Authors:** Astrid Ustad, Trine Holt Edwin, Kjerstin Næss Melsæter, Karen Sverdrup, Gro Gujord Tangen, Øystein Døhl, Pernille Thingstad, Beatrix Vereijken, Nina Skjæret-Maroni

**Affiliations:** ^1^Department of Neuromedicine and Movement Science, Faculty of Medicine and Health Sciences, Norwegian University of Science and Technology, Trondheim, Norway; ^2^Department of Geriatric Medicine, Oslo University Hospital, Oslo, Norway; ^3^Norwegian National Centre for Ageing and Health, Vestfold Hospital Trust, Oslo, Norway; ^4^Department of Finance, Trondheim Municipality, Trondheim, Norway; ^5^Department of Health and Welfare, Trondheim Municipality, Trondheim, Norway

**Keywords:** older adults, care services, health care utilization, trajectory modeling, physical activity, physical performance, daily physical behavior, accelerometry

## Abstract

**Introduction:**

Understanding factors that influence care service use is crucial for developing preventive strategies to maintain independence among older adults. In this study, we aimed to identify distinct trajectory groups of municipal care service use among community-dwelling older adults to determine whether daily physical activity is associated with future care service use.

**Methods:**

This prospective cohort study included 981 community-dwelling older adults from the HUNT4 Trondheim 70+ study. At baseline, physical activity was assessed over seven consecutive days using two accelerometers attached to the thigh and lower back. An activity type machine learning model was used to classify the physical activity types: walking, standing, cycling, running, sitting, and lying. Municipal care service use was retrieved monthly from medical records for 3 years. Using group-based trajectory modeling, we identified distinct trajectories of care service use. Multinomial regression models adjusted for age, sex, education level, dementia, and physical performance were used to evaluate the associations between daily physical activity at baseline and care service group belonging.

**Results:**

We identified four distinct trajectory groups of municipal care service use, labeled *steady low* (72.7%), *low increasing* (9.0%), *medium increasing* (12.0%), and *high increasing* (6.3%). Daily time spent in total physical activity was not associated with trajectory group belonging when adjusted for age, sex, education level, dementia, and physical performance. However, more time spent walking, in bouts lasting longer than a minute, was associated with a reduced relative risk of belonging to the *high increasing* compared to the *steady low* group. Furthermore, age, physical performance, and dementia were all significantly associated with trajectory group belonging, and sex differences were observed. Compared to women, men had a reduced relative risk of belonging to the *low increasing, medium increasing,* or *high increasing* trajectory groups.

**Conclusion:**

This study identified four distinct trajectories of municipal care service use among older adults over 3 years. Total daily physical activity was not associated with trajectories of care service use, but more time spent walking in longer bouts was independently associated with lower care service use, even when adjusted for the strong predictors of physical performance, dementia diagnosis, and age.

## 1 Introduction

Healthcare systems worldwide face significant challenges due to demographic changes, including an aging population and a decreasing proportion of working-age individuals (1). Consequently, there is a need for healthcare systems to adapt and provide sustainable care services for older adults. Care services refer to personal, social, and medical services provided at home or in institutions by professional health workers (2). Care services include a range of activities, from food delivery services to extensive long-term care provided at home and, eventually, long-term institutional care. In Norway, municipalities provide care services and long-term institutional care based on individual needs within the publicly financed welfare system. As in many other countries, the priority is to offer home care, rather than institutional care, to lower the economic cost and enable individuals to live in their own homes (1, 3, 4). The intention is that by receiving adequate care services, individuals can live at home and maintain their independence longer, which is key to the quality of life for the user and their next of kin (5).

The need for care services depends on the ability to live independently and manage daily activities, which is closely related to physical function. Changes in physical function occur across aging, and advanced age is associated with reduced cardiovascular capacity, muscle strength, balance, and flexibility (6, 7). Measures related to physical function, such as physical performance, are shown to have important clinical and practical implications for detecting functional decline (8). Identifying functional decline at an early stage is crucial, as evidence suggests it can be stabilized or even reversed with targeted interventions among community-dwelling frail older adults (9–11), potentially reducing or delaying the need for care services. Although clinically assessed physical performance may effectively represent an individual’s capacity, it does not necessarily reflect the level of actual daily activities. Consequently, device-measured daily physical activity in free-living settings can offer complementary insights and provide valuable information for predicting care service needs.

Physical activity is defined as any bodily movement that results in energy expenditure (12). There is convincing evidence of the role of exercise in preventing disease and functional decline throughout life (13, 14). However, less is known about other aspects of physical activity, such as the role of light-intensity everyday activities in maintaining function and independence at older age. In a cross-sectional population study using device-measured physical activity, we demonstrated that daily time spent walking and standing is associated with older adults’ ability to live independently (15). However, when examining daily physical activity as a potential predicting factor for care services, it is essential to consider additional factors that are relevant for physical activity levels in this population. Older age, along with reduced physical and cognitive function, is associated with lower physical activity levels (16–22). Evidence also suggests that sex and education level influence physical activity, with most studies showing that men and those with higher education are more active (17, 23, 24). Given that physical activity is a modifiable lifestyle factor and a well-established determinant of physical function (25–27), its potential for promoting healthy aging should be further explored.

Effective preventive strategies that reduce and delay the need for care services are key to addressing healthcare system challenges in the coming years. In Norway, all publicly provided municipal care services are registered (28), which enables detailed research on care service use. Understanding the progression of municipal care service use, hereafter referred to as trajectories of care service use, can provide valuable insights into the characteristics of individuals within distinct trajectories. To the best of our knowledge, no other studies have conducted trajectory modeling of municipal care service use in community-dwelling older adults. Identifying these trajectories and their associated predictive factors could have significant implications for targeting interventions aimed at maintaining function and independence in older adults. Accordingly, this prospective cohort study uses validated daily physical activity measures to investigate their association with future care service use. Specifically, we aim to identify distinct trajectory groups of municipal care service use among community-dwelling older adults and determine whether daily physical activity is associated with group belonging.

## 2 Materials and methods

### 2.1 Study design and population

In this study, community-dwelling older adults from the fourth wave of Trøndelag Health Study, 70 years and older conducted in Trondheim (HUNT4 Trondheim 70+), were included. The HUNT4 Trondheim 70+ was an ancillary data collection to the HUNT4 that took place between October 2018 and June 2019. Details of the Trondheim 70+ sample have been described previously (29, 30). Briefly, the HUNT4 Trondheim 70+ consisted of 1745 participants from the Østbyen district in Trondheim. The standardized study protocol in HUNT4 70+ included clinical examinations, self-reported questionnaires, assessments of cognitive function and physical performance, and a 1-week device-based assessment of physical activity, and was described in detail previously (30, 31). Trained health personnel conducted clinical assessments either at a test station (74%), or for those who were not able to attend the test station, at home (11%) or in nursing homes (15%). Participants in HUNT4 Trondheim 70+ provided broad informed consent for future use of data. The closest proxy gave consent for individuals with reduced ability to consent.

**Figure 1:**
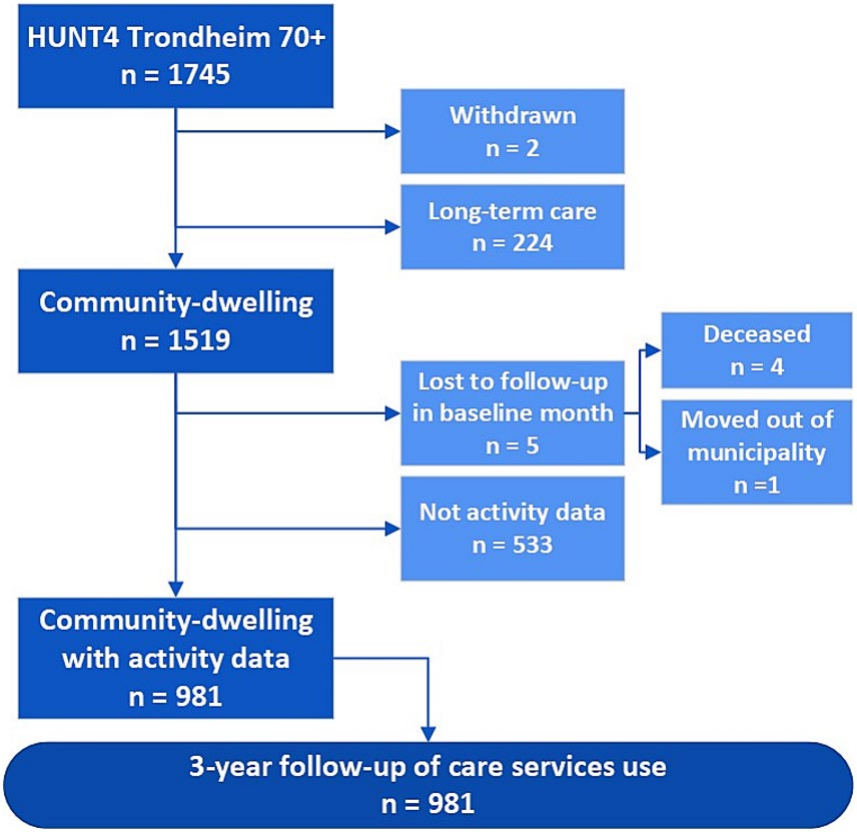
Flowchart of the HUNT4 Trondheim 70+ sample included in this prospective cohort study with three-year follow-up of municipal care service use, as well as deaths and relocation.

This prospective cohort study, with a three-year follow-up of care service use, included older adults who were community-dwelling at the time of their participation in the HUNT4 Trondheim 70+ study (*n* = 1,519, Figure 1). Of the 1,519 community-dwelling older adults, 5 participants were lost to follow-up during the month they participated in HUNT4 Trondheim 70+ (four deceased, and one moved out of the municipality). Additionally, 533 participants did not have valid activity data, resulting in 981 older adults included in this study (Figure 1.). The current study was approved by the Regional Committees for Medical and Health Research Ethics in Central Norway (ref.nr. 157470).

As reported previously, participants with valid activity data were younger, had better physical and cognitive function, and were more likely to be men compared to the participants without valid activity data (15).

### 2.2 Assessment and data analysis of physical activity

Device-based assessment of physical activity was performed using two triaxial accelerometers (Axivity AX3, Axivity, Newcastle, United Kingdom) attached to the thigh and lower back for up to 7 consecutive days. Two accelerometers were chosen, rather than a single accelerometer, because of better ability to discriminate between different types of activities (32, 33). Details regarding the configuration and positioning of the accelerometers were described previously (34). The Human Activity Recognition 70+ (HAR70+) machine learning model was used to analyze the raw accelerometer data (35). The HAR70+ model classifies the activity types walking, standing, sitting, lying, running, and cycling. The time-stamped continuous activity type classifications were post-processed using in-house Python software (Python Software Foundation, DE, United States), previously described in detail (15). In short, only days where both accelerometers were attached from the beginning to the end of the day were included, and only time awake was included in the calculation of daily averages. Time spent in the different activity types was averaged across valid days and reported in daily minutes. In addition, walking bouts and active bouts (walking, standing, running, and cycling combined) were derived. Bouts were categorized as follows: 10 to <60 s, 1 to <10 min, and ≥ 10 min. Walking bouts and active bouts were analyzed as the number of daily bouts.

### 2.3 Municipal care services

Care service use was retrieved from the medical record system in the Trondheim municipality, Norway. All public care services provided to inhabitants in the municipality are registered routinely by type of care service and the time used by the staff to provide the specific care (28). The staff registers time spent in direct contact with recipients as minutes per visit. Municipal care services include home care services (nursing care, personal care, assistance with household tasks), rehabilitation services (occupational therapy, physiotherapy, rehabilitation interventions at home), stay at daycare, stay at rehabilitation daycare, stay at rehabilitation, short-term institutional stays, and long-term institutional stays. Food service and safety alarms are also provided and registered by the municipality. In this study, received care services were retrieved monthly from the month of participation (baseline), with standardized follow-up to 34 months from the time of participation for all participants. Participants who died or moved out during the follow-up period were registered as lost to follow-up from the month of death or relocation.

For trajectory modeling of monthly care service use, care services were divided into seven pre-defined ordinal categories presented in Table 1. Categories 3–6 were defined by quartiles of the time registration of all provided care services, summed up and expressed in monthly hours. For stays at a daycare center, hours per month were computed based on the length of the stay per day and the number of days per month. For stays in short-term institutional care, where residents have 24/7 assistance, monthly hours were calculated by multiplying the number of days by 12 h per day of received care.

**Table 1:** Specification of the seven pre-defined categories used for trajectory modeling of monthly care service use.

Category		Specification
1	No care services	No care services were received in the specific month
2	Safety alarm and/or food service	No other care services were received in the specific month
3	<2.2 h/month	The thresholds for categories 3 to 6, were set as quartiles of all time-registered care services over the 35 months. Based on these thresholds, each month was classified 3 to 6 according to the total of all time-registered care services for that specific month.
4	2.2–10.1 h/month	
5	10.2–26.3 h/month	
6	>26.3 h/month	
7	Long-term institutional stay	Living in, or admitted to, a long-term institutional stay during the specific month.

### 2.4 Covariates

Using a directed acyclic graph (36), we decided which covariates to adjust for in the statistical modeling. Age, sex, education level, dementia, and physical performance were considered as covariates in the association between daily physical activity and care service use and were included in the analytical models. These covariates were selected based on their known associations with physical activity and care service use from literature and clinical reasoning. Education level was obtained from self-reported questionnaires as the highest achieved education and dichotomized to ≤13 years (primary and secondary education) and ≥ 14 years (higher education) based on the educational classification of ISCED and NUS 2000 (37). The Short Physical Performance Battery (SPPB) was used to assess physical performance. SPPB is associated with a range of adverse outcomes in older adults and is therefore used extensively in clinical settings and research (38–42). The SPPB consists of three tests: a hierarchical balance test, a 4-meter gait speed test, and a repeated sit-to-stand test, which generates a 0–12 score where higher scores indicate better physical performance (42). The cognitive status of each participant was evaluated by scientific and clinical experts using all available cognitive and clinical data from HUNT4 Trondheim 70+. This process, as well as the assessment tools and questionnaires used to diagnose dementia, were described in detail previously (30).

### 2.5 Data analysis and statistics

All statistical analyses were performed using Stata Statistical Software version 18 (StataCorp LLC, TX, United States). Baseline characteristics were reported as means ± standard deviations (SD) for numerical variables or proportions (*n*, %) for categorical variables. Differences in baseline characteristics between groups were compared pairwise using the chi-squared test for categorical data and the Mann–Whitney U test for continuous data.

Group-based trajectory modeling was applied to identify distinct trajectory groups of care service use using the Traj Stata package (43, 44). We followed Nagin and Odgers’s suggestions for model selection, meaning that decisions regarding the number and shapes of trajectory groups were guided by model fit and clinical relevance (44). All possible trajectory shapes were tested, and the goodness-of-fit of different models was estimated using the Bayesian information criterion (BIC), where values closest to zero indicate better fit. Class size was ensured to be at least 5%, the average posterior probability of group membership (APP) at least 0.7, and odds of correct classification based on the weighted posterior probability (OCC) above 5. Furthermore, trajectory groups’ confidence intervals (CIs) were controlled for overlap. Based on these criteria, we selected a four-group model with the trajectory shapes *1 1 1 2,* where progression lines *1* are linear and *2* is quadratic. Additional information regarding model selection is available in Supplementary Table S1.

To determine whether daily physical activity predicted trajectory group belonging, we used trajectory group membership as the outcome variable in multinomial logistic regression analyses. Daily physical activity variables were included separately to determine whether characteristics of daily physical activity predicted group belonging. Total physical activity, walking, and standing were reported as daily minutes. Walking and active bouts were reported as the number of daily bouts. Regression models included age, sex, education level, dementia diagnosis, and physical performance. Estimates from the regression analyses were reported as relative risk ratios (RRR) with 95% CIs. Only participants with complete baseline data were included in the regression analyses (*n* = 886, 90.3%). Multicollinearity between covariates was checked before the regression analyses using Spearman’s rank correlation (all coefficients ≤0.35). Additionally, the interaction effect between daily physical activity and physical performance was considered. The significance level was set to *p* < 0.05.

## 3 Results

### 3.1 Trajectories of care service use

**Figure 2:**
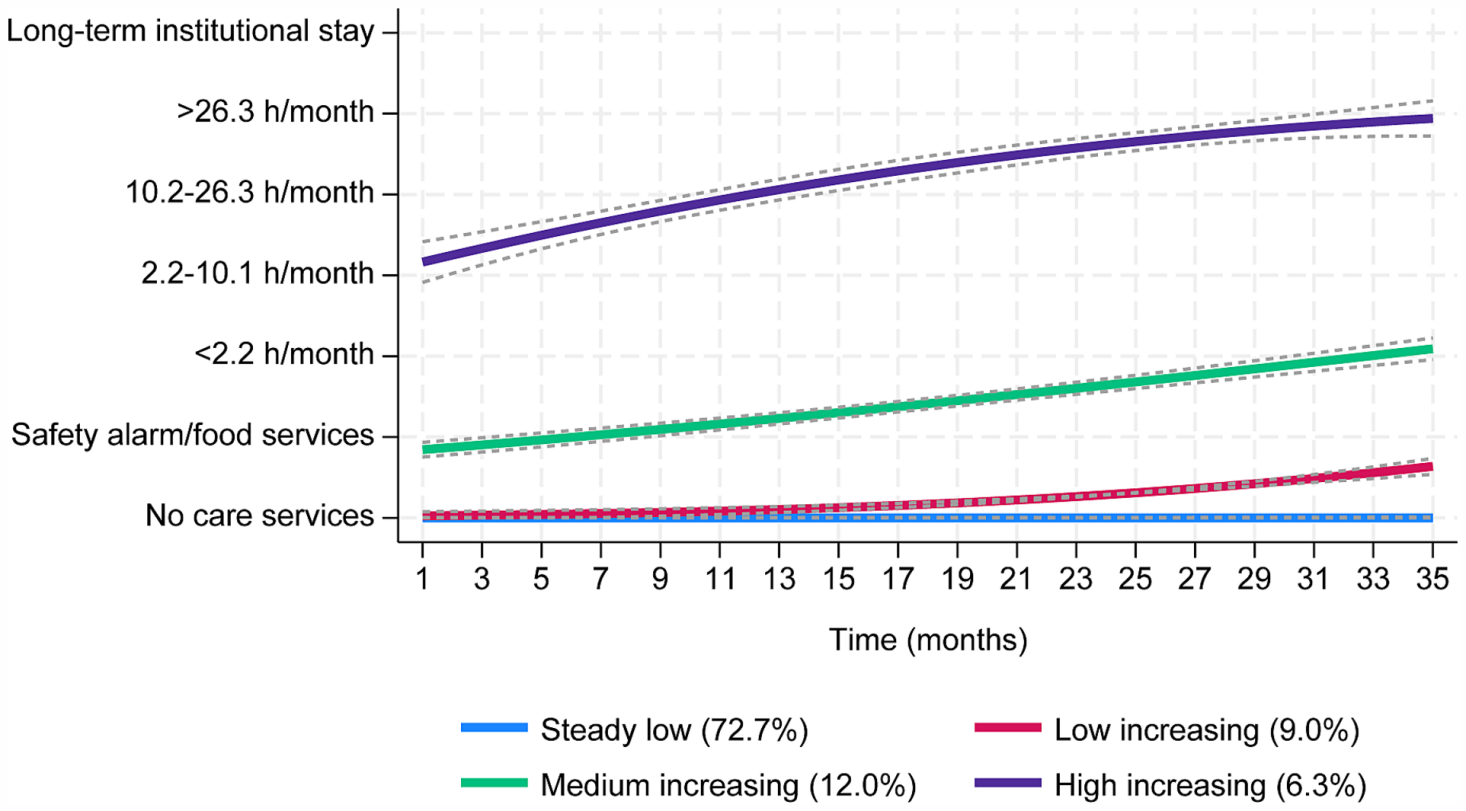
Trajectory plots of monthly care service use over 3 years among older adults that were community-dwelling at baseline. The grey dashed lines are 95% confidence intervals. The y-axis comprises seven categories, from no care service use to increased use of care services up to long-term institutional stay. Steady low group (blue); *n* = 713 (72.7%), APP = 1.00, OCC = 128.7. Low increasing group (red); *n* = 88 (9.0%), APP = 0.99, OCC = 839.7. Medium increasing group (green); *n* = 118 (12.0%), APP = 1.00, OCC = 157.6. High increasing group (purple); *n* = 62 (6.3%), APP = 0.99, OCC = 2621.9.

**Figure 3:**
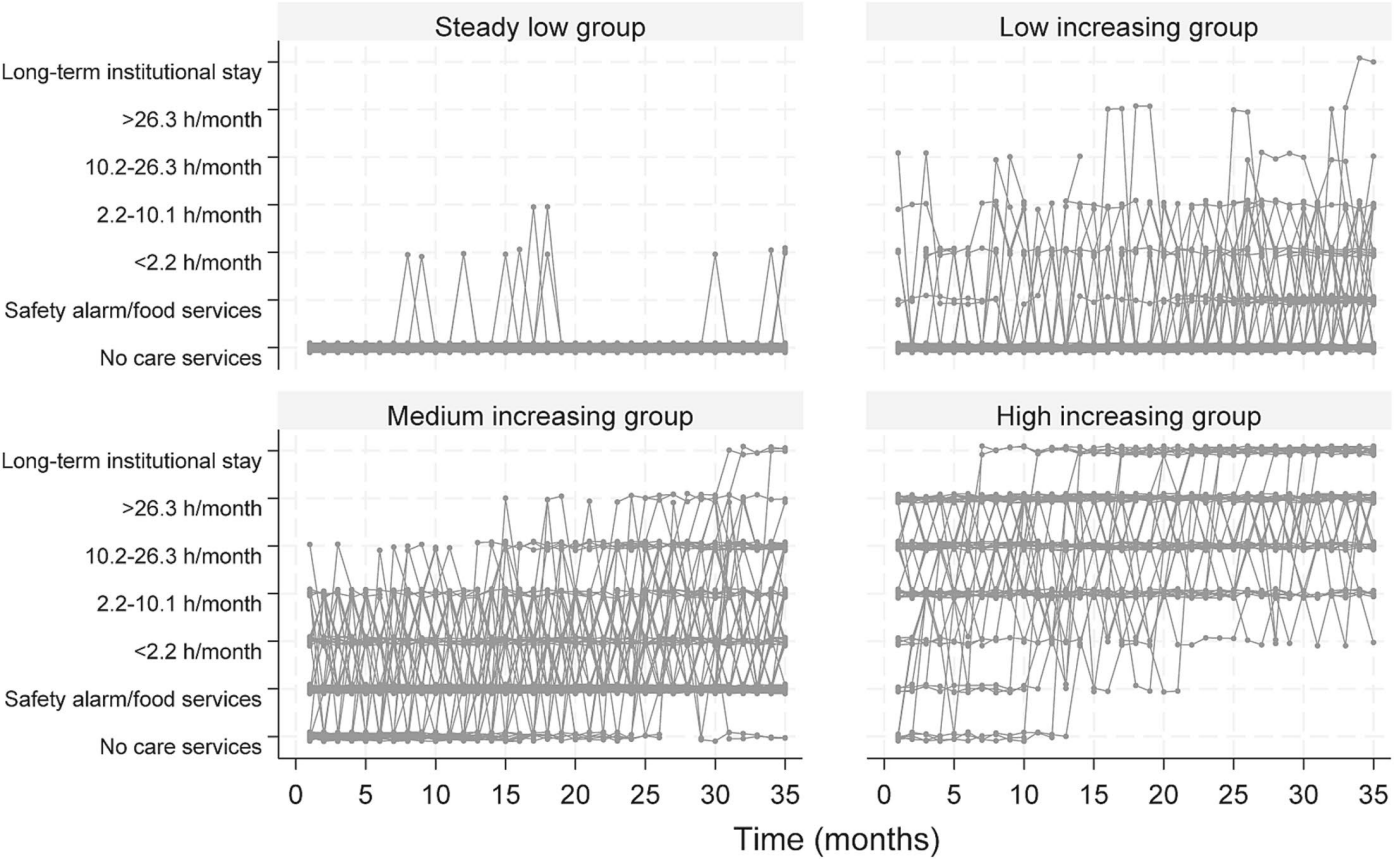
Individual trajectories for the four trajectory groups presented in Figure 2, based on monthly care service use over 3 years. The y-axis comprises seven categories, from no care service use to increased use of care services up to long-term institutional stay.

Four distinct trajectory groups of care service use were identified: *steady low* (72.7%), *low increasing* (9.0%), *medium increasing* (12.0%), and *high increasing* (6.3%) (Figure 2). Most participants belonged to the *steady low* group (72.7%), receiving no or minimal care services during the three-year follow-up period. The *steady low* groups’ trajectory was characterized by either no increase or only brief periods of increased care service use, which reverted to zero throughout the follow-up period (Figures 2, 3). The *low increasing* group demonstrated more variation, starting with very low care service use but increasing slightly towards the end of the period, with more participants receiving safety alarms and/or food services. In the *medium increasing* group, a large proportion (56.8%) had safety alarms and/or food services at baseline, and care service use increased throughout the follow-up period, culminating in a substantial amount of monthly care hours towards the end of follow-up. In the *high increasing* group, most participants received a substantial amount of care hours, which continued to rise throughout the follow-up period, with a large proportion (22.6%) living in long-term institutional care at the end of the study (Figures 2, 3).

### 3.2 Baseline characteristics by trajectory group

At baseline, the participant’s mean age was 76.9 years, ranging from 70 to 105 years (Table 2). Table 2 presents the baseline characteristics of the participants by trajectory group. Compared to the *steady low* group, participants in the three groups with higher care service use were older, had poorer physical performance, had a higher prevalence of dementia, and spent less time in daily physical activity (Table 2). Furthermore, the trajectory groups showed substantial differences in care service use at baseline, with a high proportion of the *high increasing* group already receiving various types of care services from the outset (Table 2).

**Table 2:** Baseline characteristics of participants in the HUNT4 Trondheim 70+ sample by trajectory group of care service use.

	Total (*n* = 981)	Steady low (*n* = 713)	Low increasing (*n* = 88)	Medium increasing (*n* = 118)	High increasing (*n* = 62)	Group differences
Age (years)	76.9 (5.5)	75.3 (4.1)	78.7 (5.4)	81.0 (5.7)	84.7 (7.2)	<0.05^all^
Women, *n (%)*	529 (53.9)	361 (50.6)	53 (60.2)	74 (62.7)	41 (66.1)	<0.05^b,c,^
Men, *n (%)*	452 (46.1)	352 (49.4)	35 (39.8)	44 (37.3)	21 (33.9)	<0.05^b,c^
Body mass index (kg/m^2^)	26.6 (4.3)	26.5 (4.0)	26.5 (4.8)	26.9 (4.1)	27.8 (7.1)	Not sig.
*Missing, n (%)*	*15 (1.5)*	*6 (0.8)*	*1 (1.1)*	*4 (3.4)*	*4 (6.5)*	
Gait speed (m/s)	1.0 (0.3)	1.1 (0.2)	1.0 (0.2)	0.8 (0.2)	0.7 (0.2)	<0.05^all^
*Missing, n (%)*	*25 (2.5)*	*14 (2.0)*	*3 (3.4)*	*3 (2.5)*	*5 (8.1)*	
SPPB (0–12)	10.4 (2.4)	11.1 (1.6)	10.2 (2.0)	8.8 (2.8)	6.0 (3.3)	<0.05^all^
*Missing, n (%)*	*38 (3.9)*	*26 (3.6)*	*5 (5.7)*	*4 (3.4)*	*3 (4.8)*	
MoCA (0–30)	23.9 (3.7)	24.6 (3.1)	23.0 (4.1)	22.4 (4.2)	19.2 (5.1)	<0.05^a,b,c,e,f^
*Missing, n (%)*	*37 (3.8)*	*18 (2.5)*	*3 (3.4)*	*9 (7.6)*	*7 (11.3)*	
Cognitive status*, n (%)*						<0.05^a,b,c,e,f^
Dementia	71 (7.3)	20 (2.8)	9 (10.3)	19 (16.5)	23 (37.1)	
*Missing, n (%)*	*8 (0.8)*	*4 (0.6)*	*1 (1.1)*	*3 (2.5)*	*0 (0.0)*	
Education level*, n (%)*						<0.05^a,b,c^
≤13 years	457 (49.3)	308 (44.4)	49 (59.0)	72 (65.5)	28 (68.3)	
≥14 years	470 (50.7)	385 (55.6)	34 (41.0)	38 (34.6)	13 (31.7)	
*Missing, n (%)*	*54 (5.5)*	*20 (2.8)*	*5 (5.7)*	*8 (0.8)*	*21 (33.9)*	
Care services, *n (%)*						
Safety alarm	118 (12.0)	0 (0.0)	2 (2.3)	67 (56.8)	49 (79.0)	<0.05^all^
Food services	21 (2.1)	0 (0.0)	0 (0.0)	5 (4.2)	16 (25.8)	<0.05^b,c,e,f^
Rehabilitation services	30 (3.0)	0 (0.0)	4 (4.6)	12 (10.2)	14 (22.6)	<0.05^a,b,c,e,f^
Daycare services	19 (1.9)	0 (0.0)	0 (0.0)	0 (0.0)	19 (30.6)	<0.05^c,e,f^
Household care	15 (1.5)	0 (0.0)	0 (0.0)	1 (0.8)	14 (22.6)	<0.05^b,c,e,f^
Personal care	32 (3.3)	0 (0.0)	0 (0.0)	2 (1.7)	30 (48.4)	<0.05^b,c,d,e,f^
Nursing care	54 (5.5)	0 (0.0)	0 (0.0)	9 (7.6)	45 (72.6)	<0.05^b,c,d,e,f^
Daily physical activity						
Total physical activity (min)	312 (104)	328 (95)	300 (109)	278 (92)	211 (135)	<0.05^b,c,e,f^
Walking (min)	102 (42)	110 (39)	100 (39)	84 (37)	46 (31)	<0.05^b,c,e,f^
Standing (min)	209 (77)	217 (73)	198 (82)	193 (69)	165 (112)	<0.05^c^
Walking bouts <1 min (nr)	68.9 (31.5)	73.3 (30.6)	68.9 (28.9)	59.5 (27.6)	35.3 (27.7)	<0.05^b,c,e,f^
Walking bouts 1–10 min (nr)	16.5 (9.7)	18.2 (9.9)	15.8 (8.1)	12.8 (6.9)	5.9 (5.0)	<0.05^b,c,e,f^
Walking bouts >10 min (nr)	1.0 (1.1)	1.2 (1.1)	1.0 (1.1)	0.8 (0.9)	0.3 (0.5)	<0.05^b,c,e,f^
Active bouts <1 min (nr)	15.7 (11.8)	16.3 (12.2)	15.2 (11.3)	13.9 (10.0)	13.3 (10.3)	Not sig.
Active bouts 1–10 min (nr)	25.5 (8.7)	25.3 (8.3)	26.4 (8.4)	25.7 (9.5)	25.0 (12.0)	Not sig.
Active bouts >10 min (nr)	9.3 (3.6)	9.7 (3.3)	8.9 (3.8)	8.3 (3.2)	6.0 (4.8)	<0.05^b,c,e,f^
Follow-up period						
Deceased	51 (5.2)	21 (2.9)	7 (8.0)	7 (5.9)	16 (25.8)	<0.05^a,c,e,f^
Relocation	10 (1.0)	9 (1.3)	0 (0.0)	1 (0.8)	0 (0.0)	Not sig.

SD, Standard deviation; SPPB, Short Physical Performance Battery; MoCA, Montreal Cognitive Assessment (67).

^a^Significant difference (*p* < 0.05) between steady low and low increasing.

^b^Significant difference (*p* < 0.05) between steady low and medium increasing.

^c^Significant difference (*p* < 0.05) between steady low and high increasing.

^d^Significant difference (*p* < 0.05) between low increasing and medium increasing.

^e^Significant difference (*p* < 0.05) between low increasing and high increasing.

^f^Significant difference (*p* < 0.05) between medium increasing and high increasing.

Values are given in mean (SD) for the variables age, body mass index, SPPB, MoCA, and daily physical activity, and proportions (*n*, %) for the other variables.

### 3.3 Daily physical activity and trajectories of care service use

**Figure 4:**
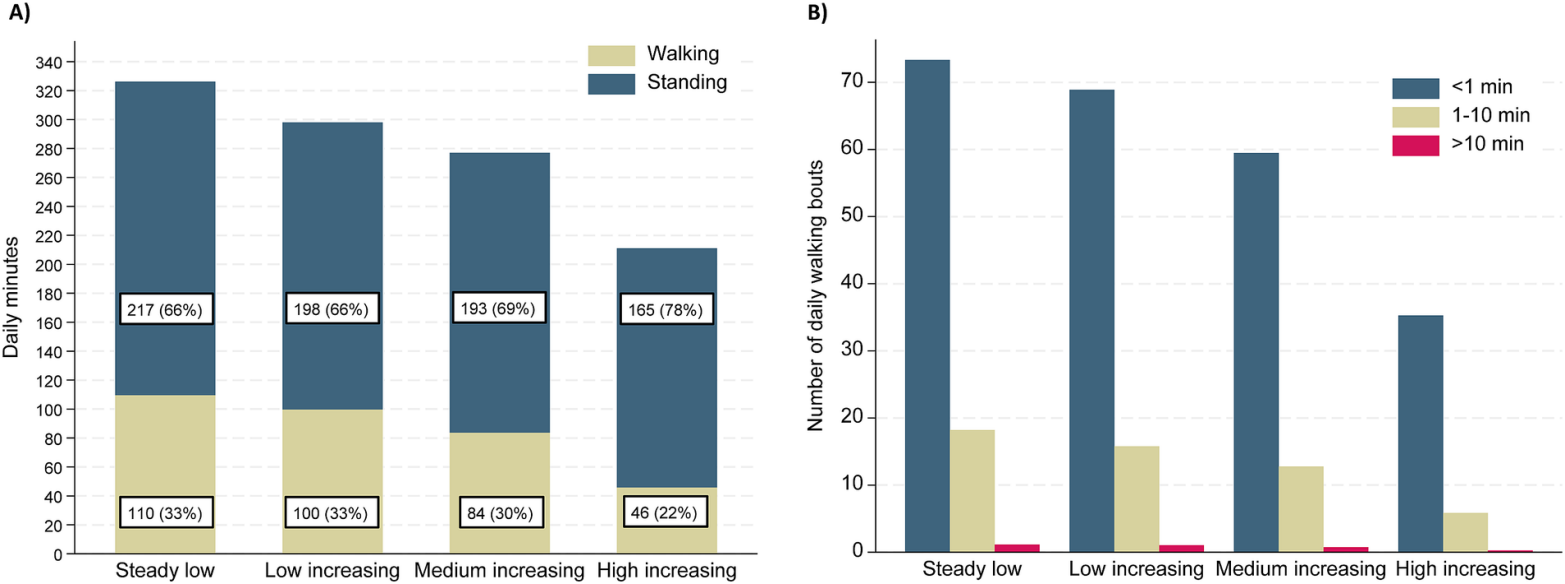
**(A)** Minutes per day spent walking and standing presented by trajectory group of care service use. **(B)** The number of daily walking bouts in categories based on duration presented by trajectory group of care service use.

Table 2 and Figure 4 show differences in daily time spent walking and standing, as well as number of walking bouts, between the trajectory groups of care service use. On average, the *steady low* group spent 1.8 h walking and 3.6 h standing, the *low increasing* group spent 1.7 h walking and 3.3 h standing, the *medium increasing* group spent 1.4 h walking and 3.2 h standing, and the *high increasing* group spent 0.8 h walking and 2.8 h standing daily. Figure 4B shows that walking bouts of all durations differed between trajectory groups, with a lower number of walking bouts in groups of higher care service usage.

The results of the multinomial regression analyses for the association between daily physical activity and trajectory group belonging are presented in Table 3. After adjusting for age, sex, education level, dementia, and physical performance, more time spent in total daily physical activity (walking, standing, cycling, and running combined) was not associated with a reduced relative risk of belonging to the low, medium, or high increasing trajectory groups compared to the steady low group (RRR 1.00, 95% CI 0.99–1.00). However, more time spent walking, particularly in bouts lasting longer than a minute, was associated with a reduced relative risk of belonging to the *high increasing* group compared to the *steady low* group. Specifically, participants with one additional walking bout lasting 1–10 min were found to have a 12% reduced risk (RRR 0.88, 95% CI 0.81 to 0.96), and those with one additional walking bout lasting longer than 10 min had a 66% reduced risk (RRR 0.34, 95% CI 0.14 to 0.83) of belonging to the *high increasing* group versus the *steady low* group.

**Table 3:** Multinomial regression models assessing trajectory group membership by daily physical activity.

	Low increasing versus steady low	Medium increasing versus steady low	High increasing versus steady low
	RRR (95% CI)	RRR (95% CI)	RRR (95% CI)
Total physical activity (min)	1.00 (0.99 to 1.00)	1.00 (1.00 to 1.00)	1.00 (0.99 to 1.00)
Age	1.15 (1.09 to 1.21) *	1.21 (1.15 to 1.27) *	1.35 (1.25 to 1.46) *
Sex	0.63 (0.38 to 1.06)	0.44 (0.26 to 0.75) *	0.19 (0.07 to 0.51) *
Education level	0.72 (0.43 to 1.21)	0.79 (0.47 to 1.32)	1.53 (0.60 to 3.91)
Dementia	4.05 (1.59 to 10.34) *	5.25 (2.15 to 12.80) *	8.92 (2.71 to 29.41) *
Physical performance	0.85 (0.74 to 0.99) *	0.67 (0.59 to 0.76) *	0.51 (0.43 to 0.62) *
Walking (min)	1.00 (0.99 to 1.00)	0.99 (0.99 to 1.00)	0.98 (0.96 to 0.99) *
Age	1.15 (1.09 to 1.21) *	1.20 (1.15 to 1.26) *	1.32 (1.22 to 1.43) *
Sex	0.68 (0.41 to 1.13)	0.46 (0.27 to 0.78) *	0.21 (0.08 to 0.57) *
Education level	0.73 (0.44 to 1.22)	0.80 (0.48 to 1.35)	1.63 (0.63 to 4.26)
Dementia	4.08 (1.60 to 10.44) *	5.23 (2.14 to 12.78) *	9.13 (2.74 to 30.40) *
Physical performance	0.84 (0.73 to 0.97) *	0.68 (0.60 to 0.77) *	0.59 (0.49 to 0.71) *
Standing (min)	1.00 (0.99 to 1.00)	1.00 (1.00 to 1.00)	1.00 (1.00 to 1.01)
Age	1.15 (1.09 to 1.21) *	1.21 (1.15 to 1.27) *	1.35 (1.25 to 1.47) *
Sex	0.62 (0.37 to 1.04)	0.45 (0.26 to 0.76) *	0.21 (0.08 to 0.57) *
Education level	0.71 (0.43 to 1.19)	0.79 (0.47 to 1.32)	1.55 (0.60 to 4.01)
Dementia	4.06 (1.59 to 10.34) *	5.31 (2.18 to 12.93) *	8.82 (2.65 to 29.35) *
Physical performance	0.85 (0.74 to 0.98) *	0.66 (0.59 to 0.75) *	0.49 (0.41 to 0.59) *
Walking bouts < 1 min (nr)	1.00 (0.99 to 1.01)	0.99 (0.99 to 1.00)	0.98 (0.96 to 1.00) *
Age	1.15 (1.09 to 1.21) *	1.21 (1.15 to 1.26) *	1.33 (1.23 to 1.44) *
Sex	0.68 (0.41 to 1.14)	0.45 (0.27 to 0.77) *	0.19 (0.07 to 0.53) *
Education level	0.72 (0.43 to 1.21)	0.80 (0.48 to 1.34)	1.62 (0.62 to 4.19)
Dementia	4.09 (1.60 to 10.44) *	5.30 (2.18 to 12.89) *	9.37 (2.83 to 31.08) *
Physical performance	0.83 (0.72 to 0.96) *	0.66 (0.59 to 0.75) *	0.55 (0.46 to 0.66) *
Walking bouts 1–10 min (nr)	0.99 (0.96 to 1.02)	0.97 (0.94 to 1.00)	0.88 (0.81 to 0.96) *
Age	1.15 (1.09 to 1.21) *	1.20 (1.15 to 1.26) *	1.32 (1.22 to 1.43) *
Sex	0.70 (0.42 to 1.17)	0.49 (0.29 to 0.83) *	0.26 (0.09 to 0.71) *
Education level	0.73 (0.44 to 1.22)	0.80 (0.48 to 1.35)	1.52 (0.58 to 3.94)
Dementia	3.99 (1.56 to 10.19) *	5.03 (2.06 to 12.25) *	8.35 (2.51 to 27.76) *
Physical performance	0.84 (0.72 to 0.96) *	0.67 (0.59 to 0.76) *	0.55 (0.47 to 0.68) *
Walking bouts > 10 min (nr)	0.99 (0.78 to 1.25)	0.89 (0.69 to 1.16)	0.34 (0.14 to 0.83) *
Age	1.15 (1.10 to 1.21) *	1.21 (1.15 to 1.27) *	1.34 (1.23 to 1.45) *
Sex	0.69 (0.42 to 1.15)	0.47 (0.28 to 0.79) *	0.21 (0.08 to 0.57) *
Education level	0.72 (0.43 to 1.20)	0.79 (0.47 to 1.32)	1.79 (0.68 to 4.66)
Dementia	4.17 (1.64 to 10.64) *	5.42 (2.22 to 13.23) *	9.63 (2.87 to 32.34) *
Physical performance	0.83 (0.72 to 0.95) *	0.66 (0.59 to 0.75) *	0.54 (0.46 to 0.65) *

min, minutes; nr, number. Total physical activity includes the activity types walking, standing, running, and cycling. Total time in physical activity, walking, and standing were reported in daily minutes of time awake. Bouts were reported as the mean number of daily bouts in categories based on duration.

Estimates are presented as Relative Risk Ratios (RRR) with 95% confidence intervals (CI).

Adjusted for age (years), sex (reference: women), education level (reference: ≤13 years), dementia (reference: no dementia), and physical performance (SPPB 0–12).

*p < 0.05, with significant effect estimates in bold.

### 3.4 Age, sex, and education level

Age was associated with trajectory group belonging, with RRRs (95% CI) of 1.15 (1.09 to 1.21), 1.21 (1.15 to 1.27), and 1.35 (1.25 to 1.46) for belonging to the *low, medium,* or *high increasing* trajectory groups, respectively, compared to the *steady low* group for each one-year increase in age. Education level had no significant associations with trajectory group belonging in this study. Compared to women, men had reduced relative risk of belonging to the *low, medium,* or *high increasing* trajectory groups with RRRs (95% CI) of 0.63 (0.38 to 1.06), 0.44 (0.26 to 0.75), and 0.19 (0.07 to 0.51), respectively.

### 3.5 Dementia and physical performance

Being diagnosed with dementia was associated with trajectory group belonging, with RRRs (95% CI) of 4.05 (1.59 to 10.3), 5.25 (2.15 to 12.8), and 8.92 (2.71 to 29.4) for belonging to the *low, medium,* or *high increasing* trajectory groups, respectively, compared to the *steady low* group.

**Figure 5:**
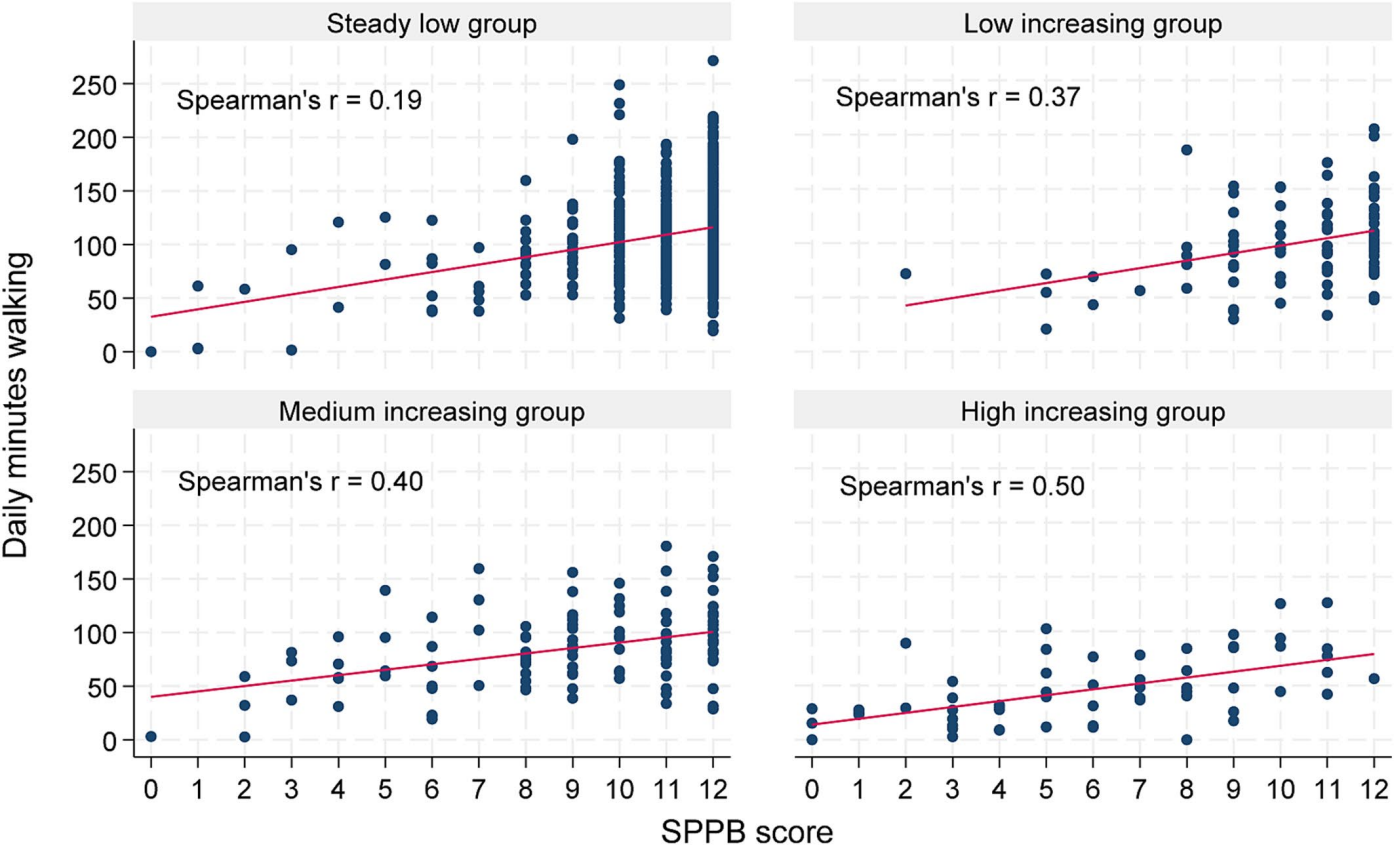
Scatter plots showing the relationship between daily minutes walking and physical performance, assessed with SPPB, presented by trajectory group of care service use. The correlation between walking and physical performance is illustrated as fitted values (in red) and reported as Spearman’s r coefficients.

Physical performance was significantly associated with trajectory group belonging. The RRRs (95% CI) for belonging to the *low, medium,* and *high increasing* groups were 0.85 (0.74 to 0.99), 0.67 (0.59 to 0.76), and 0.51 (0.43 to 0.62), respectively, for each one-unit increase in SPPB compared to the *steady low* group. No significant interaction effects between daily physical activity and physical performance were detected. There was a low overall correlation between physical performance and daily time spent walking (correlation coefficient of 0.35). However, the correlation by trajectory group of care service use revealed that this association differed between groups, with correlation coefficients of 0.19, 0.37, 0.40, and 0.50 (all *p* < 0.05) for the *steady low* group, *low increasing* group, *medium increasing* group, and *high increasing* group, respectively (Figure 5).

## 4 Discussion

This study identified distinct trajectory groups of municipal care services over 3 years among community-dwelling older adults. To the best of our knowledge, no other studies have conducted trajectory modeling of care services retrieved from medical records in community-dwelling older adults. Our findings show that physical performance, dementia, age, and sex were all strongly associated with care service use trajectories. In contrast, total time in daily physical activity was not associated with trajectory group belonging when adjusted for physical performance, dementia, age, sex, and education level. However, bouts of walking, particularly in longer bouts, were associated with a reduced relative risk of belonging to the trajectory group with the highest care service use.

### 4.1 Trajectories of care service use

The distribution of individuals across the four groups indicates that most older adults in our cohort live independently, as evidenced by 72.7% of the sample receiving no or a minimal amount of care services during this period. The individuals in the *steady low* group were younger, engaged in more daily physical activity, had a lower prevalence of dementia, and had better physical performance than those in the other trajectory groups. The mean age in the *steady low* group was close to 80 years by the end of the follow-up, suggesting that many older adults can maintain independence into advanced older age. This is in line with Huang et al. (45) who found that most older adults in China did not exhibit disability in activities of daily living when followed over 12 years.

The *low increasing* trajectory group (9.0%) generally received a low level of care services throughout the follow-up period. This group consisted of individuals who were older, had poorer physical performance, and higher prevalence of dementia compared to the *steady low* group. However, they did not differ significantly from the *steady low* group in level of daily physical activity and care service use at baseline. Over the period of 3 years, several participants in this group intermittently received various care services, primarily rehabilitation services such as physiotherapy and occupational therapy. Individuals who belonged to this specific trajectory group, had their care needs addressed by care workers, as evidenced by an increase in the use of care services. This increase was followed by a decrease, indicating their ability to return to a lower level of care. This pattern suggests that they were able to regain their independence, making them well-suited for the implementation of long-term preventive measures. Such measures, including social and exercise activities provided by the municipality, could effectively support their continued independence and reduce the strain on the healthcare system. This ability to regain independence provides a solid foundation for these individuals to enjoy additional years of life in good health.

The individuals in the *medium increasing* group (12%) exhibited trajectories characterized by an initial substantial amount of received care services that increased throughout the follow-up period, or a more rapid rise in received care services starting from no care services at baseline. This group appears to be more heterogeneous concerning the progression of care services than the other groups. In line with prioritizing home care (3, 4), these older adults received numerous care services, spanning from physiotherapy to home nursing care several times a day, reflecting the high threshold for admittance to long-term institutional care. Being able to slow down the rapid rise in care service use for the individuals in this trajectory group would be valuable for both the individual and society. Crocker et al. (46) reported that home care recipients were less likely to be admitted to long-term institutional care when receiving a multifactorial intervention, such as medication review, nutrition, and exercise, suggesting that interventions can be effective. The individuals belonging to the *medium increasing* trajectory group may be well suited to implement such interventions, which are invaluable for municipalities in preventing or delaying admission to long-term institutional care.

Finally, the participants in the *high increasing* group (6.3%) were typically older, had lower levels of daily physical activity, and the prevalence of dementia was higher (37%) compared to the other trajectory groups. This group’s trajectories were characterized by a high amount of care services received at baseline, which continued to rise, resulting in a large proportion of individuals being deceased (25.8%) or in long-term institutional care (22.6%) by the end of the study.

Several studies have used trajectory modeling to explore the patterns of physical activity (47, 48), physical performance (7, 49–51), and cognitive function (52–54) among older adults. Hu et al. (47) identified physical activity trajectories in adults over 45 years, finding that a decline in physical activity had a negative effect on cognitive function in women and on physical function in men over a three-year period. Cheng et al. (52) investigated trajectories of physical disability and cognitive function over 7 years, suggesting that reduced physical function accelerated cognitive decline in older adults. Additionally, Ikonen and colleagues (55) found that poorer physical functioning trajectories were associated with higher specialized healthcare use, highlighting the importance of public health interventions targeted at maintaining physical functioning in older age. These findings align with our results, showing that poorer physical performance was associated with trajectories of more care service use. However, none of these previous studies investigated the association with care service use. Identifying distinct trajectories of care service use among older adults can help target interventions aimed at maintaining function and independence. To achieve this, the next step could involve a closer examination of what characterizes individuals in the *steady low* group, representing those who maintain independence despite increasing age. Huang et al. (45) found that engaging in housework, a form of light-intensity physical activity, helped prevent worsening of disability over 12 years. Although our study is limited by assessing several variables at baseline only, it does provide indications of the characteristics that might contribute to maintaining independence. Consequently, there is a need for prospective studies that repeatedly assess physical activity, cognitive function, physical performance, and care service use to reveal more fully the associations between these factors.

### 4.2 Daily physical activity

A central question in the current study was whether measures of daily physical activity could provide complementary information for predicting care service use. Surprisingly, we found no association between total daily physical activity and use of care services. Previous studies have reported that physical activity and exercise are considered important lifestyle behaviors promoting healthy aging, and key therapeutic strategies of common geriatric syndromes (56–60). In a previous study, we found that standing and walking are essential for independence and participation in daily life, and constitute the primary components of everyday physical activity among older adults (15). Other studies have also established that physical activity is associated with a range of beneficial health outcomes in adults, regardless of how long, how intensive, and how often [e.g., (61)].

Numerous earlier studies have shown that physical performance, cognitive function, age, sex, and education are associated with level of physical activity (16–24). However, the relation between physical performance and daily physical activity is complex. A high level of physical activity inherently demands good physical function, while physical activity itself is a well-established determinant of physical function (26, 27). Before conducting the regression analyses in this study, we checked multicollinearity between variables, showing a low correlation between the mentioned factors and measures of daily physical activity. The highest correlation was found between daily time spent walking and physical performance, although still considered a low correlation (coefficient 0.35). Interestingly, investigating the same correlation by trajectory group of care service use revealed that the correlation between time spent walking and physical performance was low in the *steady low* group (coefficient 0.19), which constituted the majority of the sample (72.7%) and lived independently throughout the follow-up period. In contrast, the correlation between time spent walking and physical performance was much higher in the *high increasing* group (coefficient 0.50). Based on this, the *high increasing* group was closest to the expected pattern, with those having poor physical function performing fewer minutes of daily walking and those with better physical function performing more daily walking. This was evident in all trajectory groups. However, those with good physical performance showed a large variation in time spent walking, resulting in declining correlation coefficients for the *medium increasing* (0.40), *low increasing* (0.37), and *steady low* (0.19) groups. For individuals with good physical function, additional factors beyond capacity likely determine their physical activity levels. Older adults’ opportunities and decisions to engage in regular physical activity are shaped by several factors, including support from family and friends, access to exercise and recreational facilities, and personal attributes such as motivation, self-efficacy (the belief in one’s ability to execute specific actions), and self-regulation skills, such as setting achievable goals and consistently monitoring physical activity (62, 63). van Lummel et al. (64) also reported that physical performance was moderately associated with physical activity in daily life for older adults and suggested that having the capacity to be active does not mean that this capacity is used. Hence, a low level of daily physical activity in individuals with good physical performance is likely related to diverse personal, social, and environmental factors (65). Based on our findings, we suggest that the association between daily physical activity and care service use may be more related to the capacity to engage in physical activity than to the actual level of daily physical activity itself (64, 65).

There is convincing evidence that exercise has a crucial role in preventing disease and functional decline throughout life (13, 14). However, less is known about how light-intensity daily physical activity, such as walking and standing, contributes to maintaining function and independence as we age. Furthermore, the impact of exercise and daily physical activity during the lifespan on functioning in older age remains underexplored. In the current study, daily physical activity, physical performance, and other covariates were all assessed at baseline, limiting our ability to consider the impact of earlier physical activity on the function assessed at participation in HUNT4 Trondheim 70+. Longitudinal studies are needed to assess physical activity throughout the course of life to reveal the impact of daily physical activity on care service use in older age. Additionally, intervention studies examining the long-term effects of increasing daily physical activity would be valuable. Despite these limitations, it is well-established that physical activity is a modifiable lifestyle factor essential for healthy aging (1, 20, 25–27, 66), and this study provides evidence that the amount of daily walking is associated with care service use. Therefore, encouraging community-dwelling older adults to increase their time spent walking as part of everyday life should be central to public health interests.

### 4.3 Strengths and limitations

This study has several strengths. First, this study used three-year medical record data, wherein all care services provided by the municipality are registered monthly, making it suitable for conducting trajectory modeling. Second, the use of device-measured activity data, classified into activity types with high accuracy using a validated machine learning model, provided accurate measures of daily physical activity (34). Finally, the study also benefits from the diversity of the HUNT4 Trondheim 70+ sample concerning age, and physical and cognitive function.

However, some limitations of this study must be acknowledged as well. In this study, daily physical activity, physical performance, and other covariates were all assessed at a single time point (baseline), limiting our ability to account for the potential influence of physical activity earlier in life on the function evaluated at participation in HUNT4 Trondheim 70+. Furthermore, of 1,519 community-dwelling older adults who participated in the HUNT4 Trondheim 70+ study, 533 did not have activity data, and an additional 10% lacked complete baseline covariate data, and thus were excluded from the regression analyses. Consequently, the analytical sample may represent a healthier subset compared to the general population aged 70 years and older (15). Additionally, while group-based trajectory modeling is a valuable tool for identifying patterns over time in care service use, there are some limitations to the methods. Individuals are assigned to trajectory groups based on the highest posterior probability, but there could be uncertainty in group membership. Care service use among older adults can be highly individualized due to varying health conditions, personal preferences, and social support systems. Therefore, the use of trajectory modeling may overlook important within-group variability. Another limitation is that we did not include care services provided by others than the municipality, such as private care providers, family, and friends. However, this study aimed to investigate formal care services within the welfare system in Norway.

## 5 Conclusion

Community-dwelling older adults show distinct patterns in their use of municipal care services. The majority received no or minimal care services over 3 years indicating that many older adults can maintain their independence well into advanced age. Total daily physical activity was not associated with care service trajectories, but walking was associated with a lower relative risk of being in the highest care service trajectory group. Thus, increasing time spent walking as part of daily life could be a feasible intervention to maintain independence longer. Other characteristics, such as physical performance, dementia, age, and sex might be stronger predictors for future care service use. Understanding these care service trajectories provides a greater potential to prevent or delay the need for care services, as it allows us to study the characteristics of individuals within different trajectories and use this information to implement preventive strategies. Furthermore, better insight into care service trajectories among older adults can be valuable for policymakers regarding evidence-based resource planning.

## Data Availability

The datasets presented in this article are not readily available because data must be obtained from the HUNT database upon application. Data on municipal care service use may be obtained from the Norwegian Registry for Primary Health Care (https://helsedata.no/en/forvaltere/norwegian-institute-of-public-health/norwegian-registry-for-primary-health-care-kpr/). Requests to access the datasets should be directed to The HUNT database (https://www.ntnu.edu/hunt).
