# Supplementary material for: Daily physical activity and trajectories of care service use among older adults: the HUNT4 Trondheim 70+ study
Source: Front Public Health. 2025 Feb 18;13:1539179. doi: 10.3389/fpubh.2025.1539179 (PMC11876037; doi:10.3389/fpubh.2025.1539179)
Supplement: Supplementary file 1 [file Table_1.docx]

Supplementary Material

**Supplementary Table 1.** Overview of trajectory class selection.

| No. Classes | Trajectory shape | BIC* (N=981) | Smallest class size % | Lowest group APP | % <0.7 APP | Lowest OCC |
| --- | --- | --- | --- | --- | --- | --- |
| 3 | 1 1 1 | -1361 | 6.6 | 0.99 | 0.2 | 273.4 |
| 3 | 2 2 2 | -1359 | 6.6 | 0.99 | 0.2 | 254.6 |
| 3 | 3 3 3 | -1359 | 6.6 | 0.99 | 0.2 | 584.2 |
| 4 | 1 1 1 1 | -1272 | 6.3 | 0.99 | 0.3 | 149.3 |
| 4 | 2 2 2 2 | -1270 | 6.3 | 0.98 | 0.4 | 127.6 |
| **4** | **1 1 1 2 **** | **-1270** | **6.2** | **0.99** | **0.0** | **128.7** |
| 4 | 1 1 2 2 | -1301 | 4.4 | 0.98 | 0.4 | 277.7 |
| 4 | 1 1 1 3 | -1271 | 6.3 | 0.99 | 0.0 | 128.7 |
| 4 | 2 2 3 3 | -1270 | 6.3 | 0.99 | 0.4 | 123.2 |
| 4 | 2 2 1 3 | -1269 | 6.2 | 0.98 | 0.6 | 179.8 |
| 4 | 3 3 3 3 | -1272 | 6.3 | 0.99 | 0.2 | 160.4 |
| 5 | 1 1 1 1 1 | -1218 | 4.3 | 0.99 | 0.2 | 146.0 |

Group-based trajectory modeling was applied for the enumeration process. Note that all possible trajectory shapes were tested for four classes. The selection of trajectories was guided by the model fit.

Abbreviations: 1: linear progression lines, 2: quadratic progression lines, 3: cubic progression lines, BIC: Bayesian information criterion, N: number of participants, APP: average posterior probability of group membership, % <0.7 APP: Proportion of participants with individual APP <0.7, OCC: odds of correct classification based on the weighted posterior probability.

* In traj-package, the negative BIC values closest to zero indicate better fit.

** The selected model.
